# Multi‐Omics and Extracellular Vesicles: Editorial on the Research Topic

**DOI:** 10.1002/pmic.70114

**Published:** 2026-02-25

**Authors:** David W. Greening, Alin Rai, Natalie Turner

**Affiliations:** ^1^ Baker Heart and Diabetes Institute Melbourne Victoria Australia; ^2^ Baker Department of Cardiometabolic Health University of Melbourne Melbourne Victoria Australia; ^3^ Baker Department of Cardiovascular Research Translation and Implementation La Trobe University Melbourne Victoria Australia; ^4^ The Scripps Research Institute La Jolla California USA

Extracellular vesicles (EVs) represent an important mode of intercellular communication and cell regulation, by selective packaging and transfer of biological information between cells. The advent of quantitative mass spectrometry (MS)‐based proteomics, lipidomics, and various other sequencing and profiling technologies, in conjunction with advances in molecular cell biology, imaging, EV purification strategies, single EV analysis, and informatics and data integration strategies [1], has contributed significantly to our improved characterization and understanding of the molecular composition and functionality of EVs [2]. Indeed, various key studies have explored the impact of multi‐omics on EVs that have led to advanced strategies to decipher conserved EV biological markers from human cohorts that precisely differentiate EVs, selecting specific types of EVs based on EV physical features, cell‐of‐origin studies to understand EV source, multi‐biomarker identification approaches to improve diagnostic pipelines and prediction tools, single EV analytics to define disease‐specific subpopulations, and the systematic biological heterogeneity of EVs—crucial knowledge in enhancing fundamental understanding of EVs and their clinical potential.

This seminal issue, published in *Proteomics*, focuses on how multi‐omic technology and applications are advancing the characterization and refinement of EVs. Collectively, these studies signify a move toward high‐definition multi‐omics, where the focus shifts from merely identifying EV presence to quantifying the intricate molecular architecture of specialized subpopulations, workflows in understanding molecular composition, curated repositories, and integrated analytical strategies. This progression is driven by advances in MS applied to lipids and proteins, bioinformatic deconvolution, and a refined focus on specific molecular features, including membrane lipids, RNAs, and post‐translational protein signatures. The field is developing and applying expansions in technology and applications of multi‐omics to include larger datasets, novel omics layers (e.g., glycans, noncoding RNAs like lncRNAs and circRNAs, refined targeted technologies (targeted MS), spatial proteomics, and single‐vesicle analytics), and enhanced AI for predictive modelling and data integration. Such advances will impact how we understand EV composition and function. Such knowledge is the intersection of the biological complexity of EVs, and experimental limitations and analytical scalability of how multi‐omics is applied and complemented by rigorous, standardized frameworks. We thank all contributors to this special issue research topic and the referees for their prompt and in‐depth reviews.

## 1 EV Multi‐Omics

The review by Dellinger and colleagues (10.1002/pmic.70066) provides a comprehensive roadmap for transitioning from isolated, single‐modality studies to integrated, systems‐level investigations of EVs, and how advances in mass spectrometry (MS), next‐generation sequencing, and bioinformatics to characterize the diverse molecular cargo of EVs have contributed as major drivers of this transition. Through such integration of these data with clinical information and advanced computational modelling, the review illustrates how researchers can take a systems view and achieve a unified understanding of EV biogenesis, heterogeneity, and potential function(s). Within, there are discussions on how advanced analytical and computational strategies can overcome traditional multi‐omics challenges in data integration, standardization, data compatibility, and how various machine learning and AI‐assisted and network tools are being used to engineer targeted EVs for drug delivery and design of EV‐neoantigens for selective immune cell activation and potential for immunotherapy. Challenges are discussed in the context of further requirements in EV/technical standardization, reproducibility, and developments in computational and experimental tools.

## 2 Heterogeneous EVs

Several studies provide new insights by looking beyond the traditional “EV” classification. Shafiq et al. (10.1002/pmic.70051) identify a class of EVs termed “midbody remnants” (MBRs) in the context of cell polarity. The proteomic study describes a large‐scale purification procedure for obtaining highly purified MBRs and small EVs, combined with DIA‐centric proteomic analyses, to reveal that MBRs are reprogrammed during epithelial‐mesenchymal transition (EMT) and oncogenic transformation, carrying unique markers like CD73 that are absent in smaller EVs. Further, the study details the distinct protein signatures that distinguish both EV types dependent on cell source (origin), in addition to a conserved “core” MBR proteome, and further highlights the key, selective features of the MBR proteome linked with epithelial plasticity, migration, and pro‐tumorigenic transformation.

Similarly, Suzuki et al., (10.1002/pmic.70054) demonstrate that large EVs (lEVs) in ovarian cancer patients contain unique diagnostic and prognostic protein biomarkers not found in their smaller counterparts. This multi‐source proteomic analysis focused on specific EV proteins from tissue, serum, and ascites from patients with high‐grade serous ovarian cancer and concurrently separated small EVs and lEVs through sequential multistep centrifugation. Key targets identified were further associated with gene expression levels in ovarian cancer (relative to normal tissue), correlating expression in lEV proteins for both ovarian tissue and ascites‐derived samples.

In‐depth subpopulation characterization and analysis of EVs was explored by Lässer and colleagues (10.1002/pmic.70096), which employed density‐cushion separation to identify four distinct EV subtypes from human mast cells and monocytes. Subtypes included large EVs and small EVs of low and high density. Since few markers currently exist to differentiate subtypes of EVs, this study aimed to determine proteomic and lipidomic differences among these EV subpopulations, and performed further correlation using flow nanoAnalyzer. Their findings highlight that while a “core proteome” exists, specific lipid and protein enrichments (such as ceramides in large EVs) differentiate these populations based on size and density, likely reflecting diverse biogenesis pathways and specialized functions. The study demonstrated that large and small EV subpopulations of different densities are fundamentally different, a feature prominent for the proteome than the lipidome.

The transition toward “tissue‐specific” EV analysis is a major insight. Hao and colleagues (10.1002/pmic.70081) report a key advance in proteomic characterization of EVs isolated directly from prostate cancer tissue. The study identified that tissue‐derived EVs (isolated from 20 mg of tissue through an enzymatic approach combined with ultracentrifugation and density‐based purification) reflect local metabolic reprogramming (e.g., glycolysis and amino acid biogenesis) more accurately than circulating EVs, which are often diluted by the systemic environment.

## 3 A Focus on Technical Analytics and Rigor

Several studies within highlight the need for improved analytical rigor. Jensen et al. (10.1002/pmic.70043) review how advances in the analysis of EV RNA and deconvolution approaches are now used to understand cell type‐specific transcriptomes—a process known as transcriptome deconvolution. This perspective details key insights into such methodologies, including cell type deconvolution, proteome‐based deconvolution, and using different regression methods, which can potentially improve biomarker identification and understanding of EV biology (i.e., cell type‐specific EV abundances).

Technical optimization is further explored by Rafat and colleagues (10.1002/pmic.70103), whose comparison of tandem mass tag (TMT)‐based workflows against label‐free quantification (LFQ) demonstrates that TMT (a peptide‐centric isobaric labelling approach to enable multiplexed proteomics) may provide superior proteomic coverage and sensitivity, particularly in detecting subtle functional signatures in irradiated cells. However, considerations in the mode of acquisition, utility of advances in data independent acquisition for refined EV proteome coverage, and differences in separation dimensionality (extensive fractionation applied to TMT‐centric approach) likely enhanced the sensitivity and dynamic range of such TMT‐based proteome workflow. These factors need to be carefully considered with respect to the often‐limited amounts of purified EV material, while balancing proteome coverage, missingness, and analytical depth to ensure reproducible detection of low‐abundance proteins. However, the TMT pipeline applied to EVs does underscore its capacity for robust multiplexing and improved reproducibility, highlighting key EV functional implications.

The viewpoint by Nakayasu and colleagues (10.1002/pmic.70036) reviews various methods of EV isolation and purification from complex matrices. Focusing on blood plasma, they discuss methodological principles, advantages, and limitations, while contributing to refining various analytical approaches in understanding EVs, including a combined meta‐analysis approach. The challenge of using MS‐based proteomics for EV analyses for both discovery and targeted proteomics is provided. This viewpoint focuses on the potential to integrate multiple advanced methods and computationally assess EV composition independent of sample purity. Advances in various omic‐based technologies, and a combination of developments and applications of machine learning approaches presents opportunities to overcome some of the pressing challenges in the field.

## 4 The Diversity in EV omics

In regenerative medicine, Sharma et al., (10.1002/pmic.70065) show that the therapeutic potential of mesenchymal stromal cell‐derived EVs (MSC‐EVs) can be “primed” under hypoxic conditions to enhance their regenerative and immunomodulatory capacities. Hypoxic preconditioning significantly alters EV cargo, enriching miRNAs and proteins that promote angiogenesis and tissue repair, providing insight into the differential regulatory roles of EVs from different MSC sources dependent on oxygen availability.

Schjenken and colleagues (10.1002/pmic.70088) explore the composition of seminal EVs in human male fertility, profiling their protein composition to understand how they modify sperm function and prime the female immune system for conception. By analyzing and comparing EVs from different tissues and regions within the male reproductive tract, a core seminal EV proteome was identified, and included immunoregulation and signaling response factors. The authors provide a segue to improving our understanding of the role seminal EVs play in influencing human fertility.

Dohi et al. (10.1002/pmic.70102) performed a systematic review of sequencing data in the context of mouse EV‐miRNA profiles. The authors expand upon the impact of isolation methodologies in introducing technical variability and the need to focus on standardized and well‐reported guidelines for monitoring how to isolate EVs from complex biological matrices. A systematic review of 53 studies focusing on reproducibility in miRNA expression profiles of EVs from mouse serum identified several key issues apart from methodological impacts of EV isolation, including RNA extraction protocols, and unreported confounders such as platelet contamination or blood‐handling procedures that affect variability. The review emphasized the need for technical reproducibility in EV research, and that further detail and inclusion of improved data‐sharing practices in omic research [3, 4, 5] should likewise be applied to the field of EVs. Such sentiment is a key aspect of MISEV2023 (Minimal Information for Studies of Extracellular Vesicles 2023) in the context of omics reporting guidelines [6]. For “omics” technologies applied for understanding the form, function, and biology of EVs, MISEV2023 submits specific reporting to ensure that results reflect biological reality rather than technical artifact, encouraging the public deposition of raw sequencing, proteome, and omic data to allow for meta‐analytic validation. More specific databases and resources in the field (e.g., SVAtlas [7]) have focused on integrating single‐EV datasets, which include experimental parameters, heterogeneity analyses, disease‐specific marker exploration, and built‐in analysis/clustering/visualization pipelines. The integration of multi‐omics with the study of EVs provides a systems‐level view of intercellular communication, enabling the translation of complex molecular signatures into high‐precision liquid biopsies and bioengineered therapeutic platforms for personalized medicine. Further multi‐omic developments in the field are central to understand these signaling and cell regulation particles, including their structural integrity, genesis, diversity, and signaling context that allow various molecular cargo to be delivered.
